# Long-Term Withdrawal of Anticoagulation and Antiplatelet Therapy in a HeartMate 3 Left Ventricular Assist Device

**DOI:** 10.7759/cureus.28779

**Published:** 2022-09-04

**Authors:** Kristina Menchaca, Catherine A Ostos Perez, Nemanja Draguljevic, Jennifer Paciletti, Waqas Ghumman, Cristiano Faber, Sajid Mirza

**Affiliations:** 1 Internal Medicine, University of Miami/JFK (John Fitzgerald Kennedy) Medical Center, Atlantis, USA; 2 Internal Medicine, University of Belgrade, Belgrade, SRB; 3 Cardiology, University of Miami/JFK (John Fitzgerald Kennedy) Medical Center, Atlantis, USA

**Keywords:** left ventricular assist device (lvad), pump thrombosis, bleeding, antiplatelets, anticoagulation, heartmate 3

## Abstract

A 73-year-old male with end-stage heart failure underwent insertion of the HeartMate 3 (Abbott, North Chicago, IL, USA) device. Systemic anticoagulation and antiplatelets were discontinued for 52 days due to postoperative bleeding. After hemorrhage resolution, we restarted warfarin monotherapy targeting an international normalized ratio of 1.8-2.5. Eight months later, there are no reports of pump thrombosis, thromboembolism, and bleeding.

## Introduction

Pump thrombosis and thromboembolic events are one of the most drastic complications in patients with ventricular assist devices. Efforts were made to advance left ventricular assist devices (LVADs) and minimize the occurrence of adverse events. The Multicenter Study of MagLev Technology in Patients Undergoing Mechanical Circulatory Support Therapy With HeartMate 3 (MOMENTUM 3) trial demonstrated the superiority of HeartMate 3 (Abbott, North Chicago, IL, USA) over its predecessor in regards to the removal or replacement of a malfunctioning device and two-year survival free of disabling strokes. HeartMate 3 exhibited a lower incidence of bleeding events and ischemic and hemorrhagic strokes [1].

## Case presentation

A 73-year-old man with dilated non-ischemic cardiomyopathy, left ventricular ejection fraction (LVEF) of 15%, New York Heart Association (NYHA) 4, stage D, Interagency Registry for Mechanically Assisted Circulatory Support (INTERMACS) 3, and inotrope dependent on intravenous milrinone 0.5 µg/kg/min was admitted with acute on chronic systolic heart failure. Medical history was significant for chronic systolic heart failure (non-ischemic cardiomyopathy, biventricular dysfunction, LVEF 15%, NYHA 4, stage D, INTERMACS 3), status post-cardiac resynchronization therapy, paroxysmal atrial fibrillation, obstructive sleep apnea, and gastroesophageal reflux disease.

His vital signs on presentation were sinus tachycardia of 110 beats/min, blood pressure of 90/60 mmHg, respiratory rate of 18 breaths/minute, and saturation of 94% on room air. The physical exam was significant for jugular venous distention of 18 cm H_2_O, sustained hepatojugular reflux, S3 gallop with 2/6 left lower sternal border holosystolic murmur, bilateral lung rales, and extensive peripheral edema with cool distal extremities.

The patient was admitted for diuresis; however, he developed end-organ dysfunction due to low-output heart failure. Given decompensation, the decision was made to insert axillary Impella 5.5 (Abiomed, Danvers, MA, USA), a temporary left ventricular assist device. The patient was medically optimized and subsequently underwent HeartMate 3 left ventricular assist device (LVAD) implant as destination therapy. Given biventricular dysfunction, a decision was made for the right ventricular assist device (RVAD) with Protek Duo (CardiacAssist, Inc, Pittsburgh, PA, USA) at the time of LVAD implant.

**Figure 1:**
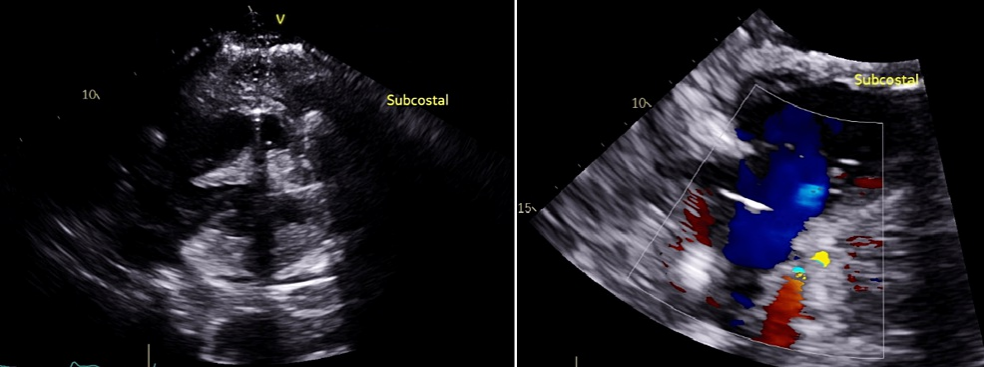
Bedside TTE. V: urgent transthoracic bedside echocardiogram, TTE: transthoracic echocardiogram.

The patient was noted to have low-flow alarms on postoperative day 1 due to acute blood loss anemia. A bedside transthoracic echocardiogram (TTE) was performed to rule out cardiac tamponade (Figure 1); the study was negative for significant pericardial effusion but did show marked thickening of the pericardial fat layer. Systemic anticoagulation and antiplatelet therapy were held, and the patient was taken for operative re-exploration. It was found that the existing left internal jugular catheter had perforated the blood vessel, and the infused fluid had dissected the pericardium with infiltration of the pericardial fat. The catheter was removed without further bleeding.

During postoperative day 2, the patient developed hypoxia, and a chest radiograph showed complete opacification of the lung field. Emergent bronchoscopy revealed diffuse alveolar hemorrhage in the left lung with thrombus occluding the left mainstem bronchus. Hemostasis was attempted with saline, epinephrine mixture, and ice-cold saline. Due to hypercapnia with respiratory acidosis, an oxygenator was added to RVAD to ventilate the right lung. The patient required inotropic and vasopressor support with dobutamine, epinephrine, norepinephrine, and vasopressin. Due to recurrent left lung hemorrhage, the patient underwent bronchial plugging and coiling of the bronchial artery. He had several bronchoscopies to extract the thrombus in the left main bronchus. Throughout this time, anticoagulation was held, and only aspirin 81 mg was given every day.

The patient then developed continuous melena and again received multiple red blood cell transfusions. Upper endoscopy revealed non-bleeding gastric erosions, and colonoscopy demonstrated diffuse melena throughout the colon. Computed tomography angiography revealed a possible jejunal source of the bleeding, although the subsequent mesenteric angiogram was negative for any active bleeding. The patient then underwent attempted push enteroscopy; however, the operator could not advance the scope past the duodenum. Aspirin was then discontinued in addition to holding anticoagulation. The bleeding had resolved with medical treatment, including a proton-pump inhibitor and octreotide infusion.

The patient was gradually weaned off the inotropes and vasopressors. RVAD was removed after 30 days following implant and had developed multiple clots within the oxygenator before its removal. However, the LVAD remained free of thromboembolic complications; there were no power elevations on daily LVAD interrogation, and hemolysis markers remained stable. For exactly 52 days, systemic anticoagulation and antiplatelet regimen were held throughout this time due to recurrent bleeding. Subsequently, anticoagulation with only warfarin was resumed. The patient spent three months in hospital and was discharged on warfarin monotherapy with an international normalized ratio (INR) goal of 1.8-2.5. Aspirin was not restarted due to a life-threatening gastrointestinal bleed. During our monthly follow-ups after hospitalization, the patient was maintained with warfarin monotherapy targeting INR 1.8-2.5. Eight months later, he has not experienced pump thrombosis, thromboembolic complications, or bleeding. Aspirin was never restarted due to his history of life-threatening gastrointestinal bleeding.

## Discussion

One of the possible explanations for the performance of HeartMate 3 (HM3) LVAD is the unique design; it features an intrapericardial centrifugal-flow pump and a magnetically levitated rotor made to decrease the occurrence of thrombotic events. Full magnetic levitation allows 10 to 20 times wider gaps for blood flow, reducing shear stress and areas of blood stasis. In addition, the pump lining is textured with sintered titanium microspheres, which serve as a biological barrier between blood and artificial material. Lastly, HeartMate 3 also exhibits an inherent pulsatility to avoid blood stasis [2].

To avoid the potential development of fatal complications such as pump thrombosis and systemic thromboembolism, it is currently recommended to use aspirin and vitamin K antagonists to target an international normalized ratio (INR) of 2.0 to 3.0. However, there are reports of exceptionally low occurrences of pump thrombosis in the HeartMate 3, which makes it relatively “clot resistant”. The MOMENTUM 3 trial showed the absence of de novo pump thrombosis in the first six months [1]. One-year results from the Conformitè Europëenne (CE) Mark trial demonstrated an absence of hemolysis, pump thrombosis, and pump malfunction [3]. Despite design improvements, the reported benefits of HeartMate 3 were observed in the context of daily aspirin and therapeutic anticoagulation.

Minimal Anticoagulation Evaluation to Augment Hemocompatibility (MAGENTUM-1), a short-term pilot trial, proposed low-intensity anticoagulation to target lower INR of 1.5 to 1.9 investigating the feasibility and safety of this in patients with HeartMate 3 devices. The MAGENTUM-1 trial showed no increase in thromboembolic complications or pump thrombosis in closely monitored patients during the use of low-intensity anticoagulation [4]. Given the increased bleeding risk of LVAD patients, further trials should continue to address low-intensity anticoagulation in the HeartMate 3. Furthermore, the role of antiplatelet agents in a patient with LVADs is not completely understood. Although MOMENTUM 3 trial suggested that the risk of adverse events is increased without an antiplatelet regimen, there are scattered data on warfarin monotherapy and discontinuation of aspirin being associated with decreased bleeding events [5]. Our case report demonstrates the discontinuation of systemic anticoagulation for almost two months and the discontinuation of antiplatelet therapy for eight months without the development of any LVAD-related thromboembolic event. This patient case adds to a pool of data analyzing the performance of HeartMate 3 and a potential new approach to anticoagulation.

## Conclusions

In light of the significant bleeding event in the HeartMate 3 LVAD, large-scale trials targeting lower INR goals or even discontinuation of therapeutic anticoagulation and antiplatelet therapy are warranted. The learning objectives of this case are 1) to recognize the occurrence of major bleeding events in LVAD patients requiring discontinuation of anticoagulation and antiplatelet therapy, 2) to understand the HeartMate 3 pump design and its associated decreased risk of pump thrombosis and thromboembolic events, and 3) to recognize the necessity of further studies to address low-intensity anticoagulation or even cessation of warfarin therapy.
